# Calcimimetics and CaSR signaling in CKD-MBD: bone–immune–vascular crosstalk and therapeutic potential

**DOI:** 10.3389/fimmu.2026.1912252

**Published:** 2026-08-11

**Authors:** Cai-Mei Zheng, Hsuan-Chu Hsu, Yi-Chou Hou, Chien-Lin Lu, Jing-Quan Zheng, Kuo-Cheng Lu

**Affiliations:** 1 Division of Nephrology, Department of Internal Medicine, School of Medicine, College of Medicine, Taipei Medical University, Taipei, Taiwan; 2 Taipei Medical University-Research Center of Urology and Kidney, Taipei Medical University, Taipei, Taiwan; 3 Division of Nephrology, Department of Internal Medicine, Taipei Medical University Shuang Ho Hospital, New Taipei City, Taiwan; 4 Division of Nephrology, Department of Internal Medicine, Taipei City Hospital, Taipei, Taiwan; 5 College of Medicine, National Yang Ming Chiao Tung University, Taipei, Taiwan; 6 School of medicine, Fu-Jen Catholic University, New Taipei City, Taiwan; 7 Department of nephrology, Cardinal Tien hospital, New Taipei City, Taiwan; 8 Division of Nephrology, Department of Medicine, Fu-Jen Catholic University Hospital, Fu Jen Catholic University, New Taipei City, Taiwan; 9 Division of Pulmonary Medicine, Department of Internal Medicine, School of Medicine, College of Medicine, Taipei Medical University, Taipei, Taiwan; 10 Division of Nephrology, Department of Medicine, Taipei Tzu Chi Hospital, Buddhist Tzu Chi Medical Foundation, School of Medicine, Tzu Chi University, Hualien, Taiwan; 11 Division of Nephrology, Department of Medicine, Tri-Service General Hospital, National Defense Medical Center, Taipei, Taiwan

**Keywords:** calcimimetics, calcium-sensing receptor, chronic kidney disease-mineral and bone disorder, osteoimmunology, vascular calcification

## Abstract

Chronic kidney disease (CKD) disrupts mineral metabolism, skeletal remodeling, vascular homeostasis, and immune regulation, producing the systemic phenotype of CKD-mineral and bone disorder (CKD-MBD) together with chronic inflammation and impaired host defense. Osteoimmunology provides a framework for understanding how bone cells, immune cells, and vascular cells interact through shared mediators such as RANKL, osteoprotegerin, NF-kappaB, NFATc1, inflammatory cytokines, fibroblast growth factor 23 (FGF23), Wnt inhibitors, and phosphate-dependent osteogenic signaling pathways. The calcium-sensing receptor (CaSR) is a class C G protein-coupled receptor that regulates parathyroid hormone (PTH) secretion and can signal through Gαq/11, Gαi/o, Gα12/13, and beta-arrestin-dependent pathways. CaSR expression and/or calcium-sensing responses have been described in bone cells, selected immune-lineage cells, and vascular smooth muscle cells, suggesting that CaSR may participate in bone-immune-vascular communication. Calcimimetics, including cinacalcet, etelcalcetide, and evocalcet, are established CaSR-targeting therapies for secondary hyperparathyroidism in dialysis patients. Their best-validated actions are PTH suppression and improvement of calcium-phosphate biochemical control; clinical and experimental studies also support reductions in FGF23, selected bone-turnover markers, and vascular calcification measures. This review critically evaluates the pharmacology of calcimimetics and their potential osteoimmune relevance in CKD-MBD. We emphasize established clinical effects while distinguishing them from mechanistic hypotheses, including direct NLRP3 inflammasome suppression, Th17/Treg rebalancing, macrophage M1-to-M2 polarization, osteocyte connexin-43/DAMP regulation, and hematopoietic niche restoration, which remain insufficiently validated in human CKD. We propose an integrated bone-vascular-immune framework and identify biomarker-guided and precision-medicine strategies for future calcimimetic research.

## 1 Introduction

Chronic kidney disease (CKD) is a multisystem disorder in which progressive loss of renal excretory and endocrine function disrupts mineral metabolism, vascular homeostasis, skeletal remodeling, and immune regulation (1–3). The concept of CKD-related bone disease has evolved from a narrow focus on phosphate and parathyroid hormone (PTH) abnormalities to the broader entity of chronic kidney disease–mineral and bone disorder (CKD-MBD), a systemic syndrome characterized by biochemical abnormalities, altered bone turnover, defective mineralization or bone strength, and vascular or soft-tissue calcification (4). CKD is also associated with chronic low-grade inflammation and acquired immune dysfunction, including premature immune aging, impaired vaccine responsiveness, increased susceptibility to infection, and increased cardiovascular risk (3, 5, 6). These observations support the view that skeletal pathology, vascular injury, and immune dysregulation in CKD are biologically interconnected rather than independent complications (7, 8).

Osteoimmunology has established that bone and immune systems share developmental niches and regulatory pathways, with skeletal and immune cells interacting through cytokines, receptors, signaling molecules, and transcription factors (7, 9). The RANKL–RANK–OPG axis, together with downstream NF-κB and NFATc1 signaling, forms a central molecular bridge between osteoclast differentiation, immune-cell activation, and inflammatory bone loss (10, 11). Inflammatory cytokines such as IL-1β, IL-6, TNF-α, and IFN-γ further modulate bone remodeling by regulating osteoclastogenesis, osteoblast–osteoclast coupling, and immune-cell–bone-cell communication (7, 11). In the uremic milieu, disruption of osteoimmune crosstalk may contribute to abnormal bone turnover, impaired hematopoietic and immune regulation, vascular calcification, and cardiovascular complications (1).

The calcium-sensing receptor (CaSR), a class C G protein–coupled receptor originally cloned from bovine parathyroid tissue, enables parathyroid cells to sense extracellular calcium and regulate PTH secretion (12, 13). CaSR signaling involves multiple downstream pathways, including Gαq/11-mediated phospholipase C activation, intracellular calcium mobilization, protein kinase C signaling, ERK1/2 activation, Gαi/o-mediated cAMP suppression, and pathway-selective responses to endogenous or allosteric ligands (14–16). Beyond the parathyroid gland, CaSR expression and/or calcium-sensing responses have been reported in bone-related cells, monocytes/macrophages, T lymphocytes, and vascular smooth muscle cells, suggesting that CaSR may influence skeletal, immune, and vascular phenotypes in a context-dependent manner (17, 18).

Calcimimetics has transformed the management of secondary hyperparathyroidism in dialysis patients by pharmacologically enhancing CaSR activity and suppressing PTH secretion (19, 20). Cinacalcet is the prototypical oral small-molecule calcimimetic, etelcalcetide is an intravenously administered peptide calcimimetic, and evocalcet is a newer oral agent developed to maintain PTH-lowering efficacy with fewer gastrointestinal adverse events (19, 21, 22). Large clinical trials, including EVOLVE and ADVANCE, evaluated whether cinacalcet could reduce cardiovascular events or attenuate vascular and valvular calcification, but these studies did not definitively establish broad cardiovascular protection or define extra-parathyroid, PTH-independent actions of calcimimetics (23–25). Emerging experimental and translational evidence suggests that calcimimetics may influence osteoblast–osteoclast coupling, bone-turnover biomarkers, FGF23, and vascular smooth muscle cell mineralization, but direct osteoimmune effects in human CKD remain investigational (26–28).

This review discuss calcimimetics effects in CKD osteoimmune axis, with emphasis on CaSR pharmacology, cell-specific signaling, bone–immune–vascular crosstalk, clinical evidence, unresolved mechanistic questions, and biomarker-guided patient stratification.

## 2 Osteoimmunology in CKD: a molecular framework

### 2.1 Normal bone–immune crosstalk and shared molecular pathways

Bone and immune cells share the bone marrow microenvironment, where hematopoietic, mesenchymal, osteoblastic, osteocytic, endothelial, and myeloid-lineage cells interact through specialized niches, cytokines, receptors, and reciprocal signaling networks (7–9). The RANKL–RANK–OPG system represents a central molecular interface between skeletal remodeling and immune regulation, because RANKL drives osteoclast differentiation while OPG restrains this process by acting as a soluble decoy receptor (29, 30). RANKL produced by osteoblast-lineage cells and, particularly in remodeling bone, osteocytes bind RANK on monocyte/macrophage-derived osteoclast precursors, recruiting TRAF6 and activating NF-κB, MAPK, c-Fos, and NFATc1 signaling programs that induce osteoclastogenic genes, including cathepsin K, MMP-9, and integrin αvβ3 (31, 32). OPG secreted by osteoblast-lineage and stromal cells competitively binds RANKL, thereby limiting RANK activation and suppressing osteoclast differentiation and bone resorption (29, 32).

Inflammatory cytokines modulate this axis: IL-1β, IL-6, and TNF-α generally enhance osteoclastogenesis directly or indirectly through RANKL-dependent pathways, whereas IFN-γ can inhibit osteoclast formation by promoting degradation or functional disruption of TRAF6-dependent RANK signaling (33, 34). Bone marrow stromal, endothelial, and osteoblast-lineage cells also regulate hematopoietic stem and progenitor cell niches through CXCL12 and stem cell factor, while osteal macrophages support osteoblast activity and bone formation, further illustrating the bidirectional coupling between skeletal and immune compartments (35, 36).

### 2.2 CKD-specific disruption of osteoimmune signaling

In CKD, the uremic milieu disrupts bone–immune communication through interacting disturbances in phosphate retention, secondary hyperparathyroidism, oxidative stress, retained uremic toxins, chronic inflammation, and acquired immune dysfunction (3). Excess phosphate is not only a mineral abnormality but also a pro-calcific signaling stimulus in vascular smooth muscle cells, where sodium-dependent phosphate transport, particularly through Pit-1, promotes osteogenic phenotypic transition and mineral deposition (37, 38). BMP-2 further amplifies phosphate-driven vascular calcification by increasing phosphate uptake, upregulating Pit-1, inducing Runx2, and suppressing smooth-muscle contractile markers such as SM22α (39). High-phosphate exposure can also impair osteoblast-lineage cell viability and contributes to the combined skeletal fragility and vascular calcification phenotype characteristic of CKD-MBD (40, 41).

Indoxyl sulfate promotes macrophage inflammation through AhR-, NF-κB-, and MAPK-dependent signaling, increasing pro-inflammatory mediators such as TNF-α and IL-1β; importantly, one macrophage study found this effect without clear activation of the NLRP3 inflammasome (42, 43). p-Cresyl sulfate and other protein-bound uremic toxins contribute to endothelial dysfunction, oxidative stress, vascular injury, and inflammatory activation in CKD (44). Advanced glycation end-products interact with RAGE and promote oxidative stress, inflammatory signaling, and vascular calcification, thereby linking metabolic stress to vascular and skeletal complications (45, 46).

Elevated PTH promotes osteoclastogenesis by increasing RANKL and suppressing OPG expression in osteoblast-lineage cells, thereby shifting the RANKL/OPG balance toward high-turnover bone resorption (47, 48). CKD is also associated with dysregulated Wnt signaling, particularly increased circulating sclerostin, while DKK1 findings are less consistent; together, these Wnt antagonists may contribute to impaired osteoblast function and altered bone turnover (49–51). In short, CKD is associated with premature immune aging, chronic inflammation, impaired innate and adaptive immune responses, and NLRP3 inflammasome activation, which may further amplify the osteoimmune disturbance of CKD-MBD.

### 2.3 The self-amplifying bone–vascular–immune vicious cycle

To reduce overlap with Section 2.2, which focuses on CKD-specific disruption of osteoimmune signaling, this section is reserved for the self-amplifying bone–vascular–immune cycle in which these disturbances reinforce one another. Pro-inflammatory cytokines released by uremia-primed immune cells, particularly IL-6, TNF-α, and IL-1β, enhance osteoclast differentiation and bone resorption through RANKL-dependent and RANKL-cooperative mechanisms, thereby linking chronic inflammation to high-turnover skeletal remodeling in CKD-MBD (7, 34). In CKD, phosphate retention is the principal driver of calcium–phosphate overload, while increased bone resorption may further contribute to mineral release from the skeleton; together, these abnormalities favor vascular calcification and ectopic mineral deposition (41, 52). Extracellular phosphate promotes vascular smooth muscle cell osteogenic conversion through type III sodium-dependent phosphate transporters, particularly PiT-1, and through ERK1/2-dependent signaling that induces osteochondrogenic gene expression (38, 52, 53).

BMP-2 amplifies phosphate-induced vascular smooth muscle cell calcification by increasing phosphate uptake, upregulating PiT-1, inducing Runx2, and suppressing smooth-muscle lineage markers such as SM22α (39, 41). FGF23 production increases in response to phosphate retention and inflammatory stimuli, and elevated FGF23 suppresses CYP27B1-mediated 1,25(OH)_2_D synthesis, thereby impairing vitamin D–dependent mineral regulation and potentially weakening vitamin D–mediated immune functions (54–56). At high concentrations, FGF23 also exerts Klotho-independent extra-renal effects, including impaired neutrophil recruitment through FGFR2-dependent signaling and pro-inflammatory FGFR4–calcineurin–NFAT signaling in noncanonical target tissues (57–59). Disruption of bone marrow stromal niches may further impair immune competence, because CXCL12- and stem cell factor–producing stromal, endothelial, and osteoblast-lineage cells are essential for hematopoietic stem-cell and early lymphoid progenitor maintenance (36, 60).

**Figure 1:**
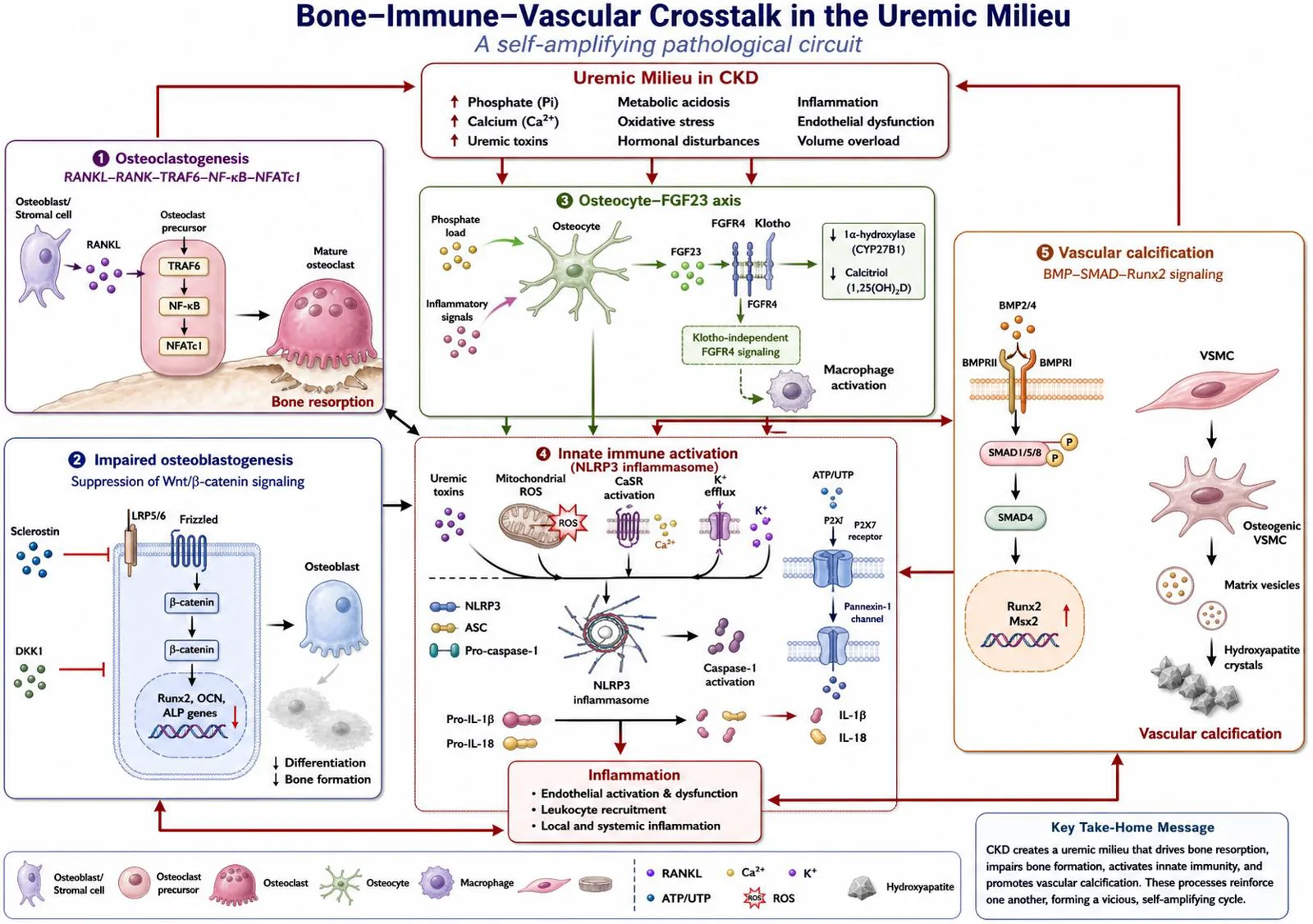
Bone–immune–vascular crosstalk in the uremic milieu. Integrated diagram showing how CKD-MBD, uremic toxins, inflammation, and vascular calcification may form a self-amplifying pathological circuit. RANKL–RANK–TRAF6–NF-κB–NFATc1 signaling promotes osteoclastogenesis, whereas suppression of Wnt/β-catenin signaling by sclerostin and DKK1 impairs osteoblast differentiation. Osteocyte-derived FGF23, induced by phosphate burden and inflammatory signaling, suppresses vitamin D activation and may promote macrophage inflammation through Klotho-independent FGFR4 pathways. Uremic toxins, mitochondrial ROS, CaSR-linked calcium signaling, K^+^ efflux, and P2X7/pannexin-1 activation converge on NLRP3 inflammasome assembly, leading to IL-1β and IL-18 release. In parallel, BMP–SMAD–Runx2 signaling drives osteogenic VSMC transition and vascular calcification.

Overall, CKD-MBD can be conceptualized as a self-amplifying bone–vascular–immune cycle in which inflammation accelerates bone resorption, mineral dysregulation promotes vascular calcification, and endocrine mediators such as FGF23 feedback on immune and cardiovascular pathways (**Figure 1**).

## 3 The calcium-sensing receptor at the osteoimmune crossroads

### 3.1 CaSR architecture and signal transduction

The calcium-sensing receptor (CaSR) is a dimeric class C G protein–coupled receptor that serves as a principal extracellular calcium sensor and regulates parathyroid hormone secretion, systemic calcium homeostasis, and additional noncalcitropic cellular functions (15, 61). Structurally, CaSR contains a large extracellular Venus flytrap domain, a cysteine-rich domain, and a seven-transmembrane domain; recent structural studies have shown that Ca²^+^, amino acids, calcilytics, and calcimimetics stabilize distinct receptor conformations within the CaSR homodimer (62, 63). Cinacalcet and evocalcet are small-molecule positive allosteric modulators that bind within the transmembrane domain and enhance CaSR responsiveness to extracellular calcium, whereas etelcalcetide is an intravenously administered peptide calcimimetic that activates CaSR through an extracellular mechanism involving disulfide interaction with Cys482 (61, 64). Therefore, rather than using ambiguous “type I/type II” terminology, calcimimetics should be classified by chemical structure, route of administration, binding site, and mode of receptor modulation (15, 61, 65). After activation, CaSR can couple to Gαq/11, Gαi/o, Gα12/13, and β-arrestin-dependent pathways, thereby regulating PLC–IP3–Ca²^+^ mobilization, cAMP suppression, ERK1/2 activation, RhoA–ROCK signaling, and receptor trafficking or desensitization (16, 61). These signaling outputs are ligand-, concentration-, receptor-conformation-, and cell-context-dependent, consistent with biased signaling and functional selectivity at the CaSR (14, 16). This signaling flexibility makes CaSR a biologically plausible target for compartment-specific modulation in CKD-MBD, although the clinical relevance of non-parathyroid CaSR signaling in bone, immune, and vascular compartments still requires further validation (15, 61).

### 3.2 CaSR expression and function in bone cells

The calcium-sensing receptor (CaSR) is expressed in skeletal cells and has been implicated in the regulation of osteoblast differentiation, osteoclast formation, osteoclast survival, bone remodeling, and mineralized matrix responses (66, 67). In osteoblast-lineage cells, CaSR activation supports calcium-dependent anabolic signaling and may promote osteoblast survival, differentiation, and mineralization through ERK1/2-, PI3K–Akt-, and Wnt/β-catenin-associated pathways (67–69). Since RANKL and OPG coordinate osteoblast–osteoclast coupling, CaSR-dependent changes in osteoblast function may indirectly influence the RANKL/OPG balance; however, specific promoter-level mechanisms involving SP1, p65 acetylation, or ERK-dependent SP1 displacement should be considered unvalidated unless supported by dedicated primary data (47, 70). In osteoclasts, extracellular calcium and CaSR-related calcium-sensing mechanisms regulate osteoclast differentiation, resorptive activity, survival, apoptosis, and cytoskeletal organization, linking local mineral concentration to feedback control of bone resorption (71–73). Osteocytes are major mechanosensory and endocrine bone cells that regulate sclerostin, RANKL, and FGF23; however, direct CaSR-mediated suppression of osteocyte-derived sclerostin or FGF23 should be presented as a proposed mechanism rather than an established effect (74–76).

### 3.3 CaSR expression and signal transduction in immune cells

The calcium-sensing receptor (CaSR) is expressed in selected immune-lineage cells, including monocytes/macrophages and human peripheral blood T lymphocytes, supporting a role for extracellular calcium sensing in immune regulation beyond systemic calcium homeostasis (77, 78). In macrophages, CaSR signaling promotes constitutive macropinocytosis through Gα-dependent, phosphatidylinositol 3-kinase, phospholipase C, Rac/Cdc42, and actin-remodeling pathways, thereby facilitating uptake of ligands for cytosolic pattern-recognition receptors such as NOD2 (77). In monocytes and macrophages, extracellular calcium can activate CaSR-dependent NLRP3 inflammasome signaling by increasing intracellular Ca²^+^ and reducing cAMP, leading to inflammasome assembly, caspase-1 activation, and IL-1β maturation (79, 80). Therefore, CaSR should be regarded as a context-dependent immune sensor that may amplify inflammatory responses under selected conditions rather than as a uniformly anti-inflammatory receptor (77, 79).

Human peripheral blood T lymphocytes express functional CaSR, and CaSR activation has been linked to MAPK- and NF-κB-dependent cytokine secretion; additional work also implies T-cell CaSR/NF-κB signaling in inflammatory cardiovascular contexts (78, 81). However, direct CaSR-dependent regulation of Th17/Treg balance, FoxP3 phosphorylation, or mTORC1 activity in CKD has not been sufficiently validated and should be described as a proposed mechanism rather than an established pathway. Dendritic cell migration and T-cell priming are strongly regulated by CCR7-dependent trafficking, but direct CaSR control of CCR7-mediated dendritic cell migration remains insufficiently proven and should be stated cautiously (82, 83).

### 3.4 The CaSR–NLRP3 paradox

**Figure 2:**
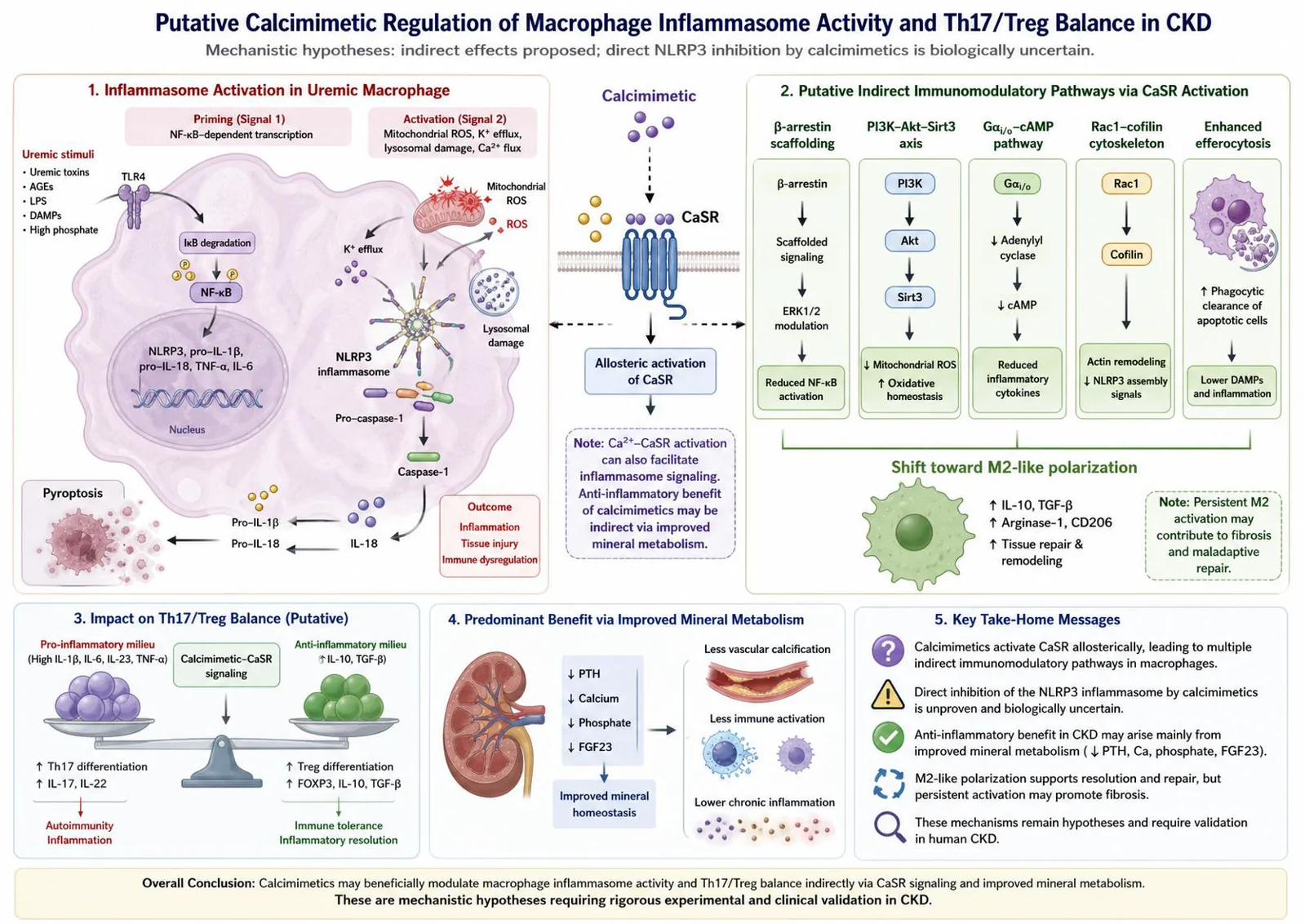
Putative calcimimetic regulation of macrophage inflammasome activity and Th17/Treg balance in CKD. Calcimimetics may influence CaSR-linked immune responses in uremia, although direct immunomodulatory effects remain poorly established. In macrophages, NF-κB-dependent priming and mitochondrial reactive oxygen species contribute to NLRP3 inflammasome activation, promoting IL-1β and IL-18 maturation and pyroptotic inflammation. Although calcimimetics activate the CaSR allosterically, proposed indirect downstream effects involving β-arrestin, PI3K–Akt–Sirt3, Gαi/o–cAMP, Rac1–cofilin, enhanced efferocytosis, M2-like polarization, and Th17/Treg rebalancing should currently be regarded as mechanistic hypotheses rather than demonstrated effects in human CKD. Because CaSR activation by extracellular calcium may also facilitate inflammasome signaling, direct calcimimetic-mediated NLRP3 inhibition is biologically uncertain. Any anti-inflammatory benefit may therefore arise predominantly from improved mineral metabolism, including reductions in PTH, calcium, phosphate, or FGF23. M2-like polarization should likewise be described as supporting inflammatory resolution and tissue remodeling, while acknowledging that persistent M2 activation may contribute to fibrosis and maladaptive repair.

**Figure 3:**
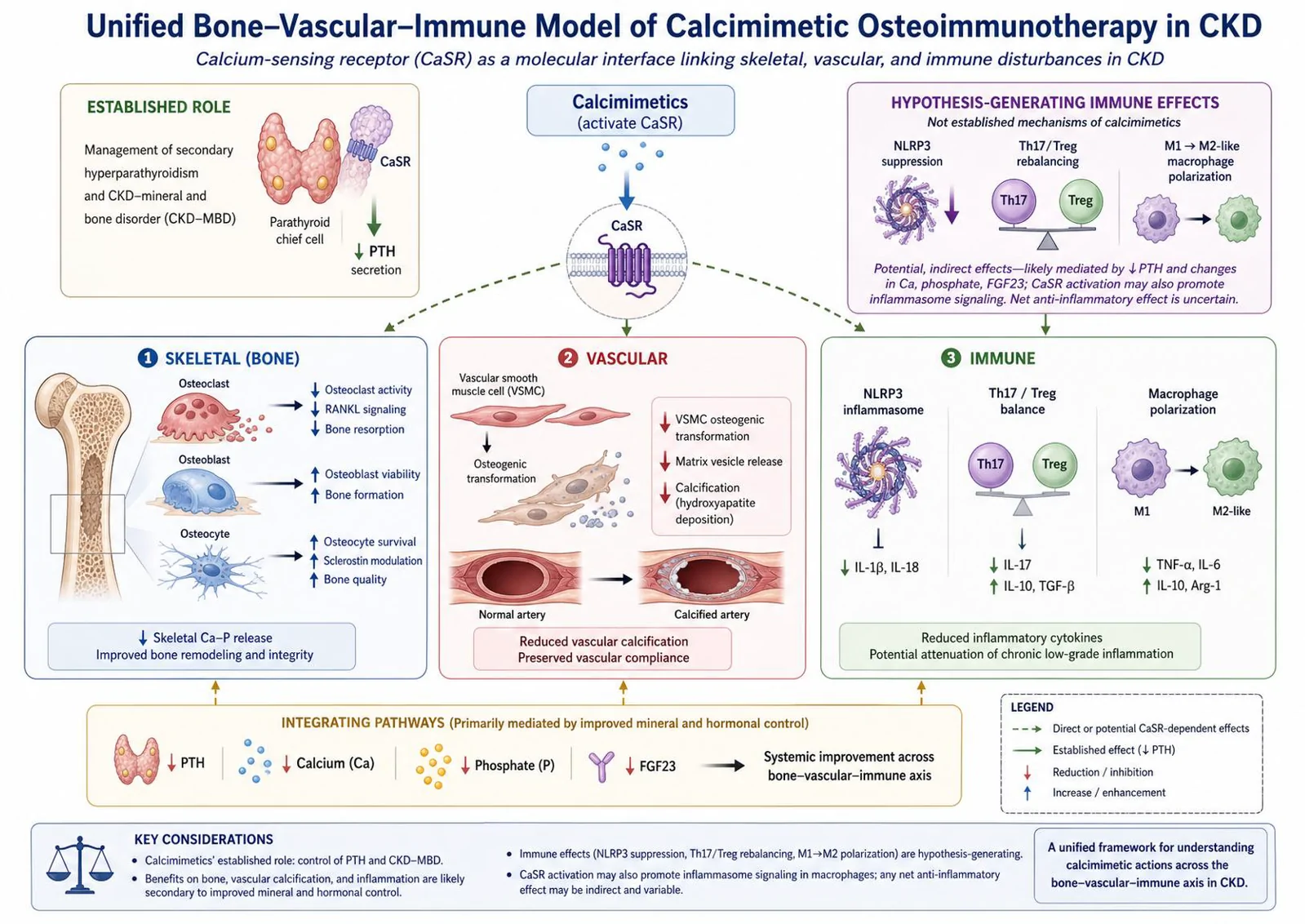
Unified bone–vascular–immune model of calcimimetic osteoimmunotherapy in CKD. This conceptual framework positions the calcium-sensing receptor as a potential molecular interface linking skeletal, vascular, and immune disturbances in CKD. By suppressing parathyroid hormone secretion—and possibly through extra-parathyroid CaSR-dependent actions—calcimimetics may limit excessive bone resorption, enhance osteoblast–osteocyte signaling, and reduce vascular smooth-muscle-cell osteogenic transformation. Their established role, however, remains primarily the management of secondary hyperparathyroidism and CKD–mineral and bone disorder. Potential increases in osteoblast viability, reductions in RANKL activity, skeletal calcium–phosphate release, inflammatory cytokines, and vascular calcification may largely reflect improved mineral and hormonal control. The proposed immune effects—including NLRP3 suppression, Th17/Treg rebalancing, and M1-to-M2-like macrophage polarization—remain hypothesis-generating and should not be presented as established calcimimetic mechanisms. Because CaSR activation may also promote macrophage inflammasome signaling, any net anti-inflammatory effect may be indirect and mediated by reductions in PTH and, variably, calcium, phosphate, or FGF23.

A conceptual tension recurs throughout this review and is addressed here explicitly. Since extracellular calcium acting through the CaSR can promote NLRP3 inflammasome assembly, caspase-1 activation, and IL-1β maturation in monocytes and macrophages, a positive allosteric modulator of the CaSR would, on strictly biochemical grounds, be expected to enhance rather than suppress NLRP3 signaling in these cells. Any proposal that calcimimetics dampen macrophage NLRP3 activity therefore cannot rest on direct CaSR agonism in immune cells; it must instead be framed as an indirect, hypothesis-generating possibility mediated by improved systemic mineral biochemistry, namely lower calcium, phosphate, PTH, and FGF23. This paradox is examined in detail in Section 6.1 and is reflected in **Figures 2** and **3**.

### 3.5 CaSR as an osteoimmune hub

The calcium-sensing receptor (CaSR) functions as a dimeric class C G protein–coupled receptor that detects extracellular calcium and can also respond to additional endogenous modulators, including magnesium, amino acids, and polyamines, as well as pharmacologic calcimimetics (15, 61). Through this broad ligand sensitivity and its capacity to signal via G proteins and β-arrestin pathways, CaSR may integrate mineral, metabolic, inflammatory, and pharmacologic signals across parathyroid, skeletal, vascular, and selected immune compartments (14, 16, 81). In vascular smooth muscle cells, CaSR expression is reduced during calcification, and experimental data show that calcimimetics can increase functional CaSR expression and reduce mineral deposition under pro-calcific conditions (17, 84). Etelcalcetide has also been shown to reduce vascular calcification in a rat model of renal insufficiency with secondary hyperparathyroidism, supporting the possibility that calcimimetics may exert vascular effects through both systemic mineral-metabolic control and direct or indirect vascular mechanisms (85). Thus, CaSR is best described as a mineral- and nutrient-sensing receptor with plausible osteoimmune and vasculoprotective relevance in CKD-MBD (15, 17, 84). However, claims that etelcalcetide produces stronger β-arrestin scaffolding, greater NLRP3 suppression, or superior anti-inflammatory effects compared with cinacalcet should be presented as hypotheses, because biased CaSR signaling is mechanistically supported but not yet validated as a clinically distinct osteoimmune effect in CKD (14, 61, 86).

## 4 Calcimimetics: pharmacology and established effects in CKD

### 4.1 Classification and molecular mechanisms

Calcimimetics are calcium-sensing receptor (CaSR)-targeting agents that enhance receptor activation and lower the extracellular calcium threshold required to suppress parathyroid hormone (PTH) secretion (15, 81). Cinacalcet is an oral small-molecule positive allosteric modulator of CaSR that increases receptor sensitivity to extracellular calcium, thereby suppressing PTH secretion in a calcium-dependent manner rather than acting as an independent orthosteric calcium agonist (19, 87).

Etelcalcetide is an intravenously administered peptide calcimimetic used in hemodialysis patients with secondary hyperparathyroidism; it activates CaSR through an extracellular disulfide-exchange mechanism involving Cys482 of the receptor (88, 89). Evocalcet is a newer oral small molecule calcimimetic that suppresses PTH with efficacy comparable to cinacalcet and fewer gastrointestinal adverse events; therefore, it should not be grouped mechanistically with etelcalcetide as a covalent extracellular peptide ligand (81, 85, 90). To summarize, since three calcimimetic agents, cinacalcet, etelcalcetide, and evocalcet differ in chemical structure, binding mechanism, route of administration, pharmacokinetics, and clinical tolerability, they might offer different biological signatures; however, clinically meaningful differences in direct osteoimmune signaling remain insufficiently proven.

### 4.2 Classical actions in secondary hyperparathyroidism

In parathyroid chief cells, activation of the calcium-sensing receptor (CaSR) suppresses parathyroid hormone (PTH) secretion by shifting the calcium–PTH response curve and lowering the calcium set-point for PTH release (15, 91). Mechanistically, CaSR activation in parathyroid tissue involves G-protein–dependent inhibition of adenylyl cyclase/cAMP signaling and activation of phospholipase C–inositol trisphosphate–intracellular calcium pathways; however, highly specific claims regarding NFAT-mediated repression of PTH transcription should be stated cautiously unless supported by dedicated primary evidence (15, 92). Cinacalcet is an oral calcimimetic that increases CaSR sensitivity to extracellular calcium and significantly lowers PTH, serum calcium, serum phosphate, and calcium–phosphate product in dialysis patients with secondary hyperparathyroidism (87, 92).

In the ACHIEVE study, cinacalcet combined with low-dose vitamin D improved achievement of biochemical treatment targets compared with flexible vitamin D therapy alone (93). Etelcalcetide, an intravenous calcimimetic administered after hemodialysis, was superior to placebo and noninferior to cinacalcet for achieving clinically meaningful PTH reduction in phase III hemodialysis trials (22). Evocalcet was noninferior to cinacalcet for PTH control in Japanese hemodialysis patients with secondary hyperparathyroidism and was associated with fewer gastrointestinal adverse events (90). In the EVOLVE trial, cinacalcet did not significantly reduce the primary composite endpoint of death or major cardiovascular events in the unadjusted intention-to-treat analysis, although adjusted and secondary analyses suggested possible benefit in selected outcomes (94). The ADVANCE study suggested that cinacalcet plus low-dose vitamin D may attenuate progression of some vascular and valvular calcification measures, but the primary coronary artery calcification endpoint should be interpreted cautiously because it did not establish definitive cardiovascular outcome benefit (23, 24) (**Table 1**). In brief, all calcimimetics through suppression of PTH secretion in secondary hyperparathyroidism by enhancing CaSR sensitivity to extracellular calcium, thereby improving biochemical control in dialysis patients, although evidence for definitive cardiovascular benefit remains limited.

**Table 1:** Summary of key preclinical and clinical evidence for calcimimetics in CKD.

Trial/study	Design	Number of patients(N)	Intervention	Primary endpoint & key findings	Osteoimmune/molecular relevance	Ref.
Phase III randomized controlled trials						
ACHIEVE study	Randomized clinical study; dialysis patients with SHPT	173	Cinacalcet plus low-dose vitamin D vs flexible vitamin D therapy	Cinacalcet plus low-dose vitamin D improved achievement of biochemical treatment targets, including PTH and calcium-phosphate parameters.	Establishes clinical PTH-lowering efficacy; FGF23 reduction was shown in related analyses/substudies, but a direct osteocyte CaSR-ERK/EGR1 mechanism remains unproven.	(27, 93, 168)
EVOLVE trial	Large randomized, double-blind, placebo-controlled trial	3883 (HD)	Cinacalcet vs placebo	The unadjusted intention-to-treat analysis did not significantly reduce the primary composite endpoint of death or major cardiovascular events; adjusted/secondary analyses suggested possible benefit in selected outcomes.	Provides clinical context for calcimimetic therapy; FGF23 reduction was associated with lower cardiovascular mortality in *post-hoc* analysis, but direct immune pathway modulation was not proven.	(28, 94, 169)
ADVANCE study	Randomized trial; hemodialysis patients with SHPT	360	Cinacalcet plus low-dose vitamin D vs flexible vitamin D therapy	Selected vascular/valvular calcification measures progressed less with cinacalcet, but the primary coronary Agatston score endpoint should be interpreted cautiously.	Supports possible anti-calcification effects, without proving a direct VSMC MGP/BMP-2/Runx2 mechanism or a vitamin K-dependent interaction.	(23, 24)
Etelcalcetide vs cinacalcet	Phase III randomized active-comparator trial	683 (HD)	IV etelcalcetide vs oral cinacalcet	Etelcalcetide was noninferior and met superiority criteria for PTH reduction compared with cinacalcet.	Confirms strong biochemical PTH control; differential beta-arrestin, NLRP3, or mTORC1 effects remain hypothetical.	(21, 65, 96)
Etelcalcetide placebo-controlled trials	Two parallel randomized placebo-controlled phase III trials	508 + 515 (HD)	IV etelcalcetide vs placebo	Etelcalcetide achieved significantly greater PTH reduction than placebo and lowered calcium, phosphate, FGF23, and bone-turnover markers.	Reductions in bone-turnover markers suggest reduced high-turnover activity but do not prove direct osteoclast apoptosis or RANKL/OPG modulation.	(22, 96, 170)
Clinical, preclinical, and mechanistic evidence						
Inflammatory biomarkers during cinacalcet therapy	Small observational/*post-hoc* studies	Variable	Cinacalcet exposure	Some studies examined CRP, IL-6, albumin, or related markers; findings are limited and heterogeneous.	Do not claim established NF-kappaB, NLRP3, or macrophage polarization effects in humans.	(150–152)
Calcimimetic effects on bone in uremic/PTH-clamped models	Preclinical CKD-MBD and PTH-clamped models	N/A	Calcimimetic therapy	Calcimimetic exposure increased osteoblast number, osteoid surface, bone formation, and osteogenic differentiation under experimental conditions.	Supports possible PTH-independent skeletal effects; not definitive human evidence.	(95)
CKD-MBD and hematopoietic niche dysfunction	Preclinical CKD-MBD mouse model	N/A	CKD-MBD model; cinacalcet tested in context	CKD-MBD impaired HSC maintenance and B-lymphopoiesis; the reported HSC defect was not restored by cinacalcet.	Supports a bone-marrow-immune link in CKD-MBD, but not calcimimetic-mediated CXCL12/SCF niche restoration.	(36, 60, 112–114)
FGF23 reduction with calcimimetics	Clinical studies and *post-hoc* analyses	Variable	Cinacalcet or etelcalcetide	Calcimimetics reduce circulating FGF23 in dialysis patients with SHPT.	FGF23 reduction is validated; the exact direct osteocyte CaSR-ERK/EGR1 mechanism remains proposed rather than proven.	(27, 28, 96, 109)

The table distinguishes validated clinical effects from exploratory mechanistic interpretations. Where reported in the original publications, sample sizes (N) are given for clinical trials and for preclinical animal or *in vitro* studies; “N/A” denotes studies for which a sample size was not reported or could not be reliably determined from the source, as indicated in the study description.

A critical appraisal of these landmark trials tempers their interpretation. In EVOLVE (94), the neutral unadjusted intention-to-treat result was strongly influenced by several features of trial design and conduct: a high rate of study-drug discontinuation, with roughly two-thirds of the cinacalcet group discontinuing treatment during the trial; substantial crossover, in which approximately one-fifth of placebo-assigned patients received commercial cinacalcet; and a baseline age imbalance, with cinacalcet-assigned patients being on average older than placebo-assigned patients. Together these factors biased the intention-to-treat comparison toward the null and help explain why prespecified age-adjusted and secondary/censoring analyses suggested a benefit that the primary analysis did not capture. For ADVANCE (23), interpretation is limited by the choice of the Agatston coronary artery calcification score as the primary metric. The Agatston method weights lesions by peak calcium density and can paradoxically increase as a plaque becomes denser and more stable, so it may register apparent progression even when calcified-plaque volume is stabilizing. Volume-based scores, which are less sensitive to mineral density, showed nominal differences favoring cinacalcet. This methodological distinction helps explain why the primary Agatston endpoint did not reach conventional significance (p ≈ 0.07) while some volume-based measures did, and it argues for cautious interpretation of the ADVANCE vascular findings.

### 4.3 Evidence for PTH-independent skeletal and vascular effects with osteoimmune implications

Experimental and translational evidence suggests that calcimimetics may exert biological effects beyond PTH suppression, because functional CaSR expression has been reported in bone cells, vascular smooth muscle cells, and selected immune-lineage cells (18, 77, 78). In vascular smooth muscle cells, CaSR protein is detectable in non-calcified vessels, CaSR expression declines during calcification, and calcimimetic exposure can reduce mineral deposition *in vitro*, supporting a potential direct vascular CaSR-dependent effect (17, 84). In uremic and PTH-clamped experimental models, calcimimetic treatment increased osteoblast number, osteoid surface, bone formation, and osteogenic differentiation despite controlled or reduced PTH exposure, indicating that some skeletal actions can occur independently of endogenous PTH secretion (95). Etelcalcetide reduced aortic calcification in a rat model of renal insufficiency with secondary hyperparathyroidism, but this effect should be interpreted cautiously because it was accompanied by reductions in serum PTH, calcium, phosphate, and FGF23 (85). Cinacalcet and etelcalcetide reduce circulating FGF23 in hemodialysis patients with secondary hyperparathyroidism, although the responsible mechanisms appear multifactorial and cannot yet be attributed solely to a direct osteocyte CaSR-ERK1/2-EGR1 pathway (96). Thus, calcimimetics should be described as established PTH-lowering agents with plausible PTH-independent skeletal and vascular actions; direct osteoimmune effects in human CKD, including macrophage or lymphocyte reprogramming, remain incompletely validated.

## 5 Potential direct effects of calcimimetics on the osteoimmune system

### 5.1 Modulation of osteoblast biology: Wnt/β-catenin and RANKL/OPG pathways

Calcimimetics may exert skeletal actions beyond parathyroid hormone suppression because CaSR expression and calcium-sensing mechanisms have been reported in osteoblasts, osteoclasts, and other bone-lineage cells, although findings vary across species, cell type, maturation stage, and experimental system (18, 97, 98). Experimental CKD models support PTH-independent anabolic skeletal effects of calcimimetic-mediated CaSR activation, including increased osteoblast number, osteoid surface, bone formation, osteogenic gene expression, and mineralization under PTH-clamped or uremic conditions (95). Through effects on osteoblast-lineage cells, CaSR activation may also influence osteoblast–osteoclast coupling by increasing OPG and reducing RANKL expression, thereby decreasing RANK-mediated TRAF6–NF-κB–NFATc1 signaling in osteoclast precursors; however, much of this mechanistic evidence derives from strontium/CaSR studies rather than clinical calcimimetic trials (70, 99).

Specific promoter-level claims, such as ERK1/2-dependent SP1 displacement from the RANKL promoter or SP1-mediated activation of the OPG promoter, should be avoided unless supported by dedicated primary data in calcimimetic-treated osteoblasts (99). CaSR-related signaling may promote osteoblast differentiation through ERK1/2-, Akt-, and Wnt/β-catenin-associated pathways, increasing osteogenic programs that include Runx2, alkaline phosphatase, osteocalcin, extracellular matrix production, and mineralization (95, 100, 101). Overall, calcimimetic/CaSR signaling can be described as a plausible dual anabolic and anti-resorptive pathway in experimental CKD-MBD, but direct osteoblast-specific and osteoimmune benefits in human CKD require further validation (95, 98, 102).

Two translational caveats apply to the *in vitro* osteoblast data cited above. First, there is a marked pharmacokinetic disparity between the concentrations used experimentally and those achieved clinically. Therapeutic oral cinacalcet doses (30–90 mg/day) produce free plasma concentrations in the low nanomolar range in hemodialysis patients, whereas many *in vitro* osteoblast and vascular smooth muscle cell studies employ micromolar concentrations (approximately 10–100 μM) (95, 99–101) that exceed clinically relevant doses, direct cellular effects may therefore not be readily translatable to patients. Second, at such supraphysiological concentrations, off-target actions become plausible: the observed anabolic responses in osteoblasts may not reflect CaSR activation alone and could involve other G-protein–coupled receptors, calcium channels, or non-specific membrane effects. Definitive attribution of these effects to the CaSR will require selective genetic approaches, such as CaSR knockdown or knockout, rather than pharmacological exposure alone (**Table 2**).

**Table 2:** CaSR expression profile and downstream signaling in bone, immune, and vascular cells in CKD.

Cell type	CaSR expression	Primary G-protein	Key downstream pathways	CKD disruption	Calcimimetic effect	Ref.
Bone Cells						
Osteoblast	High	Gαq/11; β-arrestin	PLCβ–IP3–CaM–CaMKII → proliferation; PKC–ERK1/2 → OPG↑; PI3K–Akt–GSK-3β → β-catenin stabilization → Runx2/OCN↑	↑RANKL, ↓OPG (PTH/phosphate-driven); ↓Wnt/β-catenin (sclerostin/DKK1)	May influence OPG/RANKL balance and osteoblast function in experimental settings; direct clinical evidence remains limited.	(18, 67, 70, 95, 99, 171)
Osteoclast	Moderate (precursors → mature)	Gαq/11	Calcium sensing influences NFATc1-dependent osteoclast differentiation and mature osteoclast survival/resorption; NFATc1 redirection remains hypothetical.	Hyperactivated by ↑RANKL, ↑TRAF6–IKKβ–NF-κB–NFATc1–cathepsin K axis	High extracellular calcium and strontium/CaSR models support anti-resorptive plausibility; direct cinacalcet/etelcalcetide osteoclast effects require validation.	(18, 71–73, 103–107, 172)
Osteocyte	High	Gαq/11; Gαi/o; β-arrestin	Regulates FGF23, sclerostin, RANKL, and mechanotransduction; direct CaSR control of FGF23/SOST/Cx43 remains insufficiently proven.	↑FGF23 (via IL-6–JAK-STAT/ERK); ↑sclerostin, ↑DKK1; ↑RANKL; ↑Cx43-DAMP alarmin release	Calcimimetics lower circulating FGF23; direct osteocyte CaSR-mediated suppression of FGF23, sclerostin, or Cx43-DAMP release is proposed, not established.	(74, 76, 173–175)
Immune Cells						
Monocyte (classical)	Moderate–High	Gαq/11; Gαi/o	PLCβ–IP3–ER Ca²^+^–mtROS → NLRP3–ASC–caspase-1 → IL-1β/IL-18 (Signal 2); Gαi/o–↓cAMP → ↑NF-κB → TNF-α, IL-6	NLRP3 hyperactivation by ↑Ca²^+^, phosphate, uremic toxins (TLR4/AhR)	Direct calcimimetic suppression of NLRP3 is not established; CaSR activation can promote NLRP3 in some macrophage models.	(118, 119, 176)
Macrophage activation spectrum	Moderate–High	Gαq/11; β-arrestin; Gαi/o	M1/M2 terminology is a simplified framework; macrophage states are context-dependent and shaped by cytokines, toxins, and tissue signals.	M1 dominance: IFN-γ–JAK1/2–STAT1; LPS–TLR4–MyD88–TRAF6–IKKβ–NF-κB; ↓efferocytosis	M2 polarization, efferocytosis enhancement, and CCL2 suppression by calcimimetics remain hypotheses in CKD.	(116, 117, 120)
CD4^+^ T cell (Th17)	Moderate (↑upon activation)	Gαq/11; β-arrestin	JAK1/2–STAT3 → RORγt → IL-17A/F; mTORC1 → glycolytic metabolism → Th17 maintenance	↑Th17 (uremic IL-6–JAK-STAT3); ↓mTOR-dependent glycolysis dysfunction	Indirect reduction of Th17-promoting cytokines is plausible; direct calcimimetic-mediated mTORC1 suppression or Th17 destabilization is unproven.	(78, 123, 177)
Treg (CD4^+^CD25^+^FoxP3^+^)	Moderate	β-Arrestin; Gαi/o	Treg stability depends on FoxP3, IL-2/STAT5, TGF-beta/SMAD, and metabolic pathways including mTOR; CaSR-specific effects are not established.	FoxP3 instability (↑mTORC1, ↑IL-6–STAT3 competing with SMAD2/3); Treg number and function ↓	Calcimimetic-mediated FoxP3 stabilization or Treg restoration is hypothetical and should be tested prospectively.	(78, 81, 123, 177)
Dendritic cell (myeloid)	Moderate	Gαq/11; β-arrestin	CCR7-dependent migration modulation; MHC-II and co-stimulatory molecule expression; T-cell priming (Th1/Th17 vs Treg)	Pro-inflammatory DC maturation (TLR4/NF-κB); ↑IL-12/IL-23 → ↑Th17	Direct calcimimetic induction of tolerogenic dendritic cells is not sufficiently validated.	(78, 81, 178)
Vascular Cells						
VSMC	Moderate (reduced in calcified lesions)	Gαi/o; Gαq/11; β-arrestin	CaSR is reduced during calcification; calcimimetics may reduce VSMC mineralization. Specific MGP/SP1/fetuin-A mechanisms remain proposed.	CaSR expression ↓ as calcification progresses; BMP-2–SMAD1/5/8–Runx2 ↑ (phosphate, TNF-α, IL-6)	Calcimimetics can reduce phosphate/calcium-induced VSMC mineralization *in vitro*; exact downstream mechanism remains incompletely defined.	(17, 41, 85, 179)

Stronger evidence supports CaSR relevance in parathyroid tissue, bone cells, and VSMCs; immune-cell effects of calcimimetics remain largely mechanistic or exploratory.

### 5.2 Osteoclast biology: calcium sensing, resorption control, and apoptosis

During active bone resorption, osteoclasts are exposed to high local extracellular calcium concentrations released from the mineralized matrix, and this calcium-rich microenvironment can function as a feedback signal that modulates osteoclast activity (103–105). Mature osteoclasts express CaSR mRNA, and extracellular calcium or CaSR agonists can regulate pit-forming bone resorption, supporting a role for CaSR-related calcium sensing in osteoclast feedback control (104). High extracellular calcium induces intracellular calcium responses in osteoclasts through receptor-operated, phospholipase C–linked, store-independent calcium influx pathways (105). High extracellular calcium can directly induce apoptosis in mature osteoclasts and inhibit bone resorption, providing a plausible negative-feedback mechanism during active mineral dissolution (106). Calcium signaling is also essential for osteoclast differentiation and function because calcium–calmodulin–calcineurin signaling activates NFATc1, a master transcription factor required for RANKL-induced osteoclastogenesis (73). Strontium ranelate studies provide additional evidence that CaSR activation can induce mature osteoclast apoptosis and reduce RANKL-induced osteoclast differentiation *in vitro*, supporting the plausibility of anti-resorptive effects mediated through CaSR-related pathways (72, 107). However, these data derive mainly from extracellular calcium and strontium models rather than from calcimimetic-specific osteoclast studies; therefore, direct effects of cinacalcet or etelcalcetide on osteoclast apoptosis, actin-ring disruption, or resorption-pit reduction should be described as mechanistically plausible but not yet definitively established (72, 103). Similarly, the proposed redirection of NFATc1 from cathepsin K/MMP-9 transcription toward Bim/caspase-3-dependent apoptosis, as well as a detailed PKC–p38–ATF2 apoptotic pathway, should be considered hypothetical unless supported by dedicated calcimimetic-specific primary studies (72, 73) (**Table 2**).

### 5.3 Osteocytes as endocrine, mechanosensory, and osteoimmune-regulatory cells

Osteocytes are terminally differentiated osteoblast-lineage cells embedded within mineralized bone matrix, where they act as major endocrine and mechanosensory regulators of phosphate metabolism, bone remodeling, and skeletal adaptation through production of fibroblast growth factor 23, sclerostin, RANKL, and mechanically induced signaling mediators (74, 108). As the principal bone-derived phosphaturic hormone, FGF23 is produced predominantly by osteocytes and regulates phosphate and vitamin D metabolism through fibroblast growth factor receptor–Klotho signaling in the kidney (74). Cinacalcet reduces circulating FGF23 in dialysis patients, but this effect is likely multifactorial and should not be attributed solely to a direct osteocyte CaSR–ER (27, 109). Excess FGF23 in CKD has been linked to adverse extra-skeletal effects, including Klotho-independent cardiovascular and inflammatory signaling in noncanonical target tissues (58, 59, 110). High FGF23 can impair neutrophil recruitment and host defense in CKD by interfering with chemokine-triggered β2-integrin activation, providing a mechanistic link between mineral dysregulation and innate immune dysfunction (57). Osteocyte connexin-43 hemichannels mediate mechanosensitive release of prostaglandin E_2_ and other small signaling molecules, contributing to anabolic skeletal responses to mechanical loading (111). However, direct regulation of osteocyte connexin-43 hemichannel opening by CaSR activation or calcimimetic therapy remains insufficiently validated and should be presented as a hypothesis rather than an established osteoimmune mechanism.

### 5.4 Bone marrow microenvironment and hematopoiesis

CKD-MBD can disrupt the hematopoietic stem-cell niche; in an experimental CKD-MBD model, chronic renal failure reduced bone marrow HSC frequency, impaired long-term repopulating capacity, altered HSC cell-cycle status, and was associated with osteal macrophage and B-lymphopoietic defects (112). Importantly, this study reported that the CKD-MBD–associated HSC defect could not be restored by the PTH-lowering agent cinacalcet**, i**ndicating that calcimimetic therapy should not currently be described as a proven hematopoietic niche-restorative intervention (112). CXCL12-producing stromal and endothelial niches are essential for hematopoietic stem and progenitor-cell maintenance, while osteoblast-derived CXCL12 contributes particularly to early lymphoid progenitor maintenance rather than global HSC maintenance (36, 60). Stem cell factor, also known as KIT ligand, is another key niche cytokine required for HSC maintenance and is produced mainly by endothelial and leptin receptor–positive perivascular stromal cells in bone marrow (113). Uremia can induce functional incompetence of bone marrow-derived stromal cells, providing a plausible mechanism by which CKD may impair hematopoietic support, immune competence, and reparative cell function (114).

Therefore, CKD-MBD–associated disruption of the bone marrow niche may contribute to immune dysfunction, but calcimimetic-mediated restoration of CXCL12/SCF signaling, common lymphoid progenitor output, B-cell production, or Ly6C^hi monocyte balance remains unvalidated and should be presented only as a hypothesis requiring dedicated experimental testing (36, 111, 112, 114). The osteoblast, osteoclast, osteocyte, and bone-marrow-niche mechanisms discussed in Section 5 are supported predominantly by *in vitro* and animal data—frequently using strontium or extracellular-calcium surrogates rather than calcimimetics—and none has been validated in human CKD; the corresponding levels of evidence are summarized in **Table 3**.

**Table 3:** Levels of evidence for the proposed osteoimmune and vascular actions of calcimimetics in CKD-MBD.

Proposed mechanism/effect	Level of evidence	Basis	Status in human CKD	References
PTH suppression; calcium–phosphate control	Established clinical	Randomized controlled trials (ACHIEVE, etelcalcetide and evocalcet trials; EVOLVE biochemistry)	Established	(22, 23, 93, 94, 148)
FGF23 reduction	Established biochemical effect; mechanism uncertain	Clinical studies and *post-hoc* analyses	Effect established; direct osteocyte CaSR pathway unproven	(27, 28, 96)
Attenuation of vascular/valvular calcification	Suggestive clinical	ADVANCE (secondary and volume measures; primary Agatston endpoint not significant)	Plausible but not definitive	(23, 24)
Direct VSMC CaSR-mediated anti-calcification	Experimental	*In vitro* human VSMC and rat renal-insufficiency models	Not validated in humans	(17, 84, 85)
Osteoblast anabolic action; RANKL/OPG modulation	Experimental	*In vitro* and PTH-clamped/uremic animal models (often strontium or Ca surrogates)	Hypothesis-generating	(26, 95, 100)
Osteoclast apoptosis/anti-resorption	Experimental	Extracellular-calcium and strontium models	Hypothesis-generating	(72, 102, 106)
Osteocyte Cx43/sclerostin/FGF23 regulation	Hypothesis	Indirect and experimental data	Strictly hypothetical	(74, 76, 111)
Bone-marrow niche/hematopoietic restoration	Contradicted/negative	CKD-MBD model: cinacalcet did not restore the HSC defect	Not supported	(112, 114)
Macrophage NLRP3 inflammasome suppression	Hypothesis; partly contradicted	CaSR agonism activates NLRP3 in monocytes/macrophages *in vitro*	Strictly hypothetical; biochemically counterintuitive	(79, 80, 118, 119)
Macrophage M1-to-M2 polarization	Hypothesis	Indirect experimental data; no *in vivo* genetic evidence in CKD	Strictly hypothetical	(77, 115, 186)
Th17/Treg rebalancing; FoxP3 stabilization	Hypothesis	Indirect experimental data	Strictly hypothetical	(78, 123, 177)
Reduction of CPP-induced inflammation	Hypothesis (indirect)	Untested; plausible via improved mineral biochemistry	Untested hypothesis	(119, 130)

This summary distinguishes established clinical effects, human biomarker findings, animal or *in vitro* evidence, and hypothesis-generating mechanisms, providing a uniform framework for the levels of evidence discussed across Sections 5–7. Only PTH suppression and calcium–phosphate control are established clinical effects; FGF23 reduction is an established biochemical effect of uncertain mechanism; vascular-calcification attenuation is suggestive but not definitive; and all direct osteoimmune mechanisms remain experimental or hypothesis-generating pending validation in human CKD.

## 6 Calcimimetics and immune dysregulation in CKD

### 6.1 Monocyte/macrophage polarization: NF-κB, NLRP3 inflammasome, and M1/M2 signaling

Macrophage activation is best understood as a dynamic and context-dependent spectrum rather than a rigid M1/M2 binary classification, although pro-inflammatory macrophage programs are commonly associated with TLR/MyD88/NF-κB signaling and production of IL-1β, IL-6, TNF-α, IL-12, and reactive oxygen species (115). In CKD, retained uremic toxins and chronic inflammatory stimuli promote monocyte/macrophage activation through oxidative stress, NF-κB/MAPK signaling, NLRP3 inflammasome-related pathways, and transporter-dependent toxin uptake (42, 116, 117). Indoxyl sulfate, for example, can promote pro-inflammatory macrophage activation through OATP2B1-mediated uptake and Dll4–Notch signaling, supporting a direct link between protein-bound uremic toxins and vascular inflammation in CKD (117). CaSR activation can promote NLRP3 inflammasome assembly by increasing intracellular Ca²^+^ and reducing cAMP, leading to caspase-1 activation and IL-1β maturation; therefore, CaSR should not be considered uniformly anti-inflammatory in macrophages (118, 119). Consequently, claims that calcimimetics directly suppress CaSR-dependent NLRP3 activation, induce β-arrestin–IKKϵ–Sirt3 signaling, or drive M1-to-M2 macrophage reprogramming in CKD should be presented as mechanistic hypotheses rather than established clinical effects (116, 120). Thus, calcimimetic might influence macrophage-mediated inflammation indirectly by improving CKD-MBD biochemistry, lowering PTH, modifying calcium–phosphate balance, and reducing FGF23, but direct immune-cell effects require dedicated validation in CKD models and human studies (**Table 4**).

**Table 4:** Key molecular signaling pathways in CKD osteoimmunology: disruption mechanisms and calcimimetic-relevant interpretations.

Signaling pathway/axis	Normal function	CKD disruption	Calcimimetic-relevant interpretation	Validated/proposed outcome	Ref.
Bone remodeling pathways					
RANKL–RANK–TRAF6–IKKβ–NF-κB (p50/p65)–NFATc1	Drives osteoclastogenesis; essential for bone remodeling homeostasis	↑RANKL (PTH, phosphate, IL-1β, IL-6, T cells); ↓OPG; exuberant osteoclast activation and bone resorption	Calcimimetics may indirectly normalize RANKL/OPG through PTH reduction; direct SP1 or beta-arrestin/IKKepsilon mechanisms remain unproven.	Reduced high-turnover bone resorption is plausible, mainly through PTH control and bone-turnover normalization.	(11, 70, 95)
Wnt/β-catenin–GSK-3β–TCF/LEF	Promotes osteoblast differentiation, bone formation; inhibits adipogenesis	↑Sclerostin (DMP1-driven SOST), ↑DKK1 (uremic toxins, glucocorticoids) → LRP5/6 antagonism → GSK-3β active → β-catenin degradation	CaSR-related Wnt/beta-catenin support is plausible in experimental models; direct clinical restoration by calcimimetics is not established.	Potential support of osteoblast activity; human validation remains limited.	(49–51)
CaSR–ERK1/2–FGF23/Sclerostin axis (Osteocyte)	Couples mineral sensing to hormonal regulation of phosphate metabolism	↑FGF23 (IL-6–JAK-STAT, hyperphosphatemia); ↑sclerostin → suppressed CYP27B1, impaired immune regulation	Calcimimetics reduce circulating FGF23; direct CaSR-ERK/EGR1 or SOST suppression in osteocytes remains proposed.	Strong clinical FGF23 reduction; uncertain direct osteocyte mechanism.	(76, 174, 180, 181)
Innate immune pathways					
NLRP3 inflammasome–ASC–Caspase-1–IL-1β/IL-18	Sterile danger sensing; acute host defense against pathogens	Constitutive activation by uremic Ca²^+^ (CaSR–Gαq→IP3→ER Ca²^+^), K^+^ efflux (P2X7), mtROS, indoxyl sulfate (AhR–NADPH oxidase)	Do not claim direct calcimimetic suppression; CaSR activation can promote NLRP3 activation in some models.	NLRP3 is an osteoimmune bridge; calcimimetic inhibition remains unvalidated.	(116, 118, 182)
TLR4–MyD88–IRAK1/4–TRAF6–NF-κB (Macrophage M1)	Pattern recognition; pro-inflammatory cytokine induction for microbial clearance	Chronic activation by uremic toxins (LPS-like AhR ligands, AGEs–RAGE, DAMPs from osteocytes/apoptotic cells)	Calcimimetic anti-inflammatory effects are possible indirectly through mineral control, but direct NF-kappaB suppression is not proven.	Indirect inflammatory improvement is plausible; clinical evidence is limited.	(42, 116, 117, 120)
Macrophage activation spectrum	Tissue repair, anti-inflammatory resolution, efferocytosis	Uremic serum blocks PI3K–Akt–STAT6; ↓IL-4R expression; defective efferocytosis → secondary necrosis → ↑DAMPs	Direct calcimimetic-induced M2 polarization or enhanced efferocytosis is not adequately validated.	Mechanistic hypothesis only.	(116, 120)
AhR–ARNT–OAT1 (Uremic toxin accumulation)	Xenobiotic detoxification; immune regulation through AhR-responsive genes	Indoxyl sulfate/p-cresyl sulfate activate AhR → NADPH oxidase ROS → NF-κB → IL-1β, TGF-β1–SMAD2/3 (fibrosis); OAT1↑ → ↑intracellular toxin	No strong evidence that calcimimetics suppress AhR signaling or OAT1/OATP toxin uptake.	Remove or mark as speculative.	(117, 128, 183)
Adaptive immune pathways					
JAK1/2–STAT3–RORγt (Th17 differentiation)	Host defense against fungi and extracellular bacteria; mucosal immunity	↑IL-6 (uremia) → constitutive JAK1/2–STAT3 → ↑RORγt → ↑IL-17A/F → endothelial dysfunction, ↑RANKL from T cells	Direct calcimimetic inhibition of Th17 differentiation is not validated; indirect effects through reduced inflammatory burden are possible.	Exploratory hypothesis.	(122, 123, 177)
mTORC1–FoxP3 (Treg stability)	Metabolic checkpoint governing Treg vs Th17 fate; immune tolerance	↑mTORC1 (nutrient excess, oxidative stress, ↑Rheb GTPase) → FoxP3 Ser418 phosphorylation → proteasomal degradation → Treg instability	Direct calcimimetic-mediated FoxP3 stabilization or mTORC1 suppression is not validated.	Exploratory hypothesis.	(122, 123, 177)
Vascular calcification pathways					
BMP-2–ALK2/3–SMAD1/5/8–Runx2 (VSMC osteogenesis)	Embryonic bone morphogenesis; fracture repair via periosteal cells	Hyperphosphatemia–Pit-1–ERK1/2 + TNF-α–NF-κB → ↑BMP-2 → SMAD1/5/8–SMAD4–Runx2 → VSMC transdifferentiation, TNAP, matrix vesicles	Calcimimetics reduce VSMC mineralization experimentally and may attenuate calcification clinically, but exact BMP-2/Runx2 mechanisms need validation.	Strong calcification relevance; mechanism partly unresolved.	(38, 41, 85, 146)
CaSR–Gαi/o–↓cAMP–↑MGP/CaSR–PKC–SP1–↑MGP	Endogenous vascular calcification inhibition via carboxylated MGP and fetuin-A	CaSR downregulated in calcified VSMC; loss of MGP induction; ↑uncarboxylated inactive MGP (vitamin K deficiency)	MGP/fetuin-A are important calcification inhibitors; direct calcimimetic induction/internalization mechanisms are not conclusively proven.	Supportive biology, not definitive mechanism.	(84, 143, 179)

Established effects include PTH suppression, FGF23 reduction, bone-turnover changes, and possible vascular-calcification attenuation; direct immune-restorative pathways remain hypotheses requiring validation.

#### 6.1.1 The CaSR–NLRP3 paradox in macrophages

The dominant experimental evidence indicates that CaSR agonism activates, rather than suppresses, the NLRP3 inflammasome in monocytes and macrophages. Increased extracellular calcium is a recognized danger signal that drives CaSR-dependent, Gαq/11- and Gαi/o-mediated NLRP3 assembly and IL-1β release (115). On this basis, positive allosteric modulation of the CaSR by calcimimetics could theoretically exacerbate, rather than attenuate, macrophage-driven sterile inflammation, and this possibility must be stated explicitly rather than confined to caveats. Consequently, any net anti-inflammatory action of calcimimetics in CKD is more plausibly indirect—arising from reductions in the calcium, phosphate, PTH, and FGF23 stimuli that promote inflammasome priming and calciprotein-particle formation—than from direct CaSR-mediated NLRP3 suppression within immune cells (116, 120). **Figure 2** and its legend should therefore be read as a speculative and partly contradicted model rather than as an established pathway. These mechanisms remain speculative and require further investigation.

#### 6.1.2 The dual role of M2 macrophages

M2-like macrophage polarization should not be regarded as uniformly beneficial. Although M2 programs contribute to inflammation resolution and efferocytosis, persistent or excessive M2 activation has been implicated in tissue fibrosis and maladaptive repair in chronic kidney disease. Any calcimimetic-associated shift toward M2-like phenotypes therefore carries a double-edged potential, favoring both inflammation resolution and, under chronic conditions, profibrotic tissue remodeling (121).

### 6.2 T-Cell homeostasis: JAK–STAT3, mTORC1–FoxP3, and Th17/Treg balance

Uremia is associated with adaptive immune dysregulation, including an imbalance between pro-inflammatory Th17 cells and FoxP3-positive regulatory T cells, which may contribute to persistent microinflammation and cardiovascular risk in patients with advanced CKD or maintenance hemodialysis (122, 123). IL-6 promotes Th17 differentiation by activating gp130–STAT3 signaling and inducing RORγt-dependent IL-17 programs, whereas FoxP3-positive regulatory T cells maintain immune tolerance and restrain excessive inflammatory responses (124). In CKD, elevated IL-6 and related inflammatory cytokines provide a plausible mechanistic link between uremic inflammation and Th17/Treg imbalance (122, 123). Human peripheral blood T lymphocytes express functional CaSR, and CaSR activation has been shown to stimulate ERK1/2 and NF-κB signaling with increased cytokine secretion (78). Additional experimental evidence indicates that CaSR activation can enhance T-cell apoptosis and cytokine secretion in inflammatory models, supporting a context-dependent immunoregulatory role for T-cell CaSR (125). However, direct evidence that calcimimetics restore Th17/Treg balance in CKD through CaSR–β-arrestin–Akt–TSC1/2–mTORC1 signaling, reduced FoxP3 Ser418 phosphorylation, or direct T-cell metabolic reprogramming is currently insufficient. Therefore, calcimimetic-mediated normalization of Th17/Treg homeostasis should be presented as a mechanistic hypothesis and exploratory research direction rather than an established therapeutic effect in CKD-MBD.

### 6.3 Antigen-presenting cells and NLRP3 inflammasome regulation

The NLRP3 inflammasome is a cytosolic multiprotein complex composed of the NLRP3 sensor, the adaptor protein ASC, and procaspase-1; upon activation, this complex promotes caspase-1 cleavage, maturation of IL-1β and IL-18, and gasdermin D-dependent pyroptotic cell death (126). In CKD, chronic inflammation and reduced clearance of uremic toxins contribute to persistent innate immune activation, while NLRP3 activation may be driven by mitochondrial reactive oxygen species, K+ efflux, crystal or mineral stress, and NF-kappaB-mediated priming (116, 127). Indoxyl sulfate can further promote macrophage inflammatory activation through crosstalk among aryl hydrocarbon receptor signaling, NF-kappaB, and SOCS2, thereby linking protein-bound uremic toxins to immune and vascular inflammation (43). Because IL-1β can enhance osteoclastogenic signaling and inflammatory bone resorption, NLRP3 activation provides a biologically plausible bridge between uremic inflammation, immune-cell activation, and CKD-MBD progression (7, 116). However, calcimimetic-specific inhibition of NLRP3 in antigen-presenting cells has not been firmly established. In contrast, experimental data show that CaSR activation can promote NLRP3 inflammasome activation through increased intracellular Ca2+ and reduced cAMP; therefore, proposed calcimimetic-mediated suppression of NLRP3 should be presented as a hypothesis requiring direct validation (118, 119).

### 6.4 Crosstalk with uremic toxins: CaSR, AhR, and transporter signaling

Indoxyl sulfate, a protein-bound uremic toxin retained in CKD, promotes macrophage inflammatory activation through aryl hydrocarbon receptor, NF-κB, and MAPK-dependent signaling, increasing cytokines such as TNF-α and IL-1β (42, 43). However, indoxyl sulfate–induced IL-1β production should not be automatically equated with NLRP3 inflammasome activation, because one macrophage study reported AhR/NF-κB/MAPK-mediated IL-1β induction without detectable NLRP3 inflammasome activation (42). Indoxyl sulfate also promotes vascular inflammation by entering macrophages through organic anion transporter/organic anion transporting polypeptide pathways, including OATP2B1, and by activating Dll4–Notch signaling (117). Recent reviews further support a model in which indoxyl sulfate uptake and AhR activation promote oxidative stress, NF-κB/MAPK signaling, and pro-inflammatory macrophage polarization in uremic atherosclerosis (128). p-Cresyl sulfate is another protein-bound uremic toxin associated with oxidative stress, vascular injury, cardiovascular events, and mortality risk in CKD, although its cellular mechanisms differ from those of indoxyl sulfate (129). No validated evidence currently supports the claim that calcimimetics reduce intracellular indoxyl sulfate accumulation by suppressing OAT1 transcription through a CaSR–ERK1/2–EGR1 pathway. Therefore, calcimimetics should be framed primarily as established mineral-metabolic therapies with possible indirect immunomodulatory effects (**Figure 3**).

### 6.5 Calciprotein particles: A CaSR–NLRP3 link between mineral stress and immune-mediated vascular injury

Calciprotein particles (CPPs) are fetuin-A–stabilized colloidal nanoparticles of calcium and phosphate that form in the serum of patients with CKD when the mineral-buffering capacity of fetuin-A and related inhibitors is exceeded (119, 130). CPPs have emerged as central mediators linking mineral dysregulation, inflammation, and vascular calcification in the precise clinical context this review addresses. Circulating CPP load and a reduced serum calcification propensity (a shorter T50) increase as renal function declines and have been associated with vascular calcification progression, arterial stiffness, and cardiovascular mortality in CKD (130). Mechanistically, monocytes internalize CPPs through calcium-induced, CaSR-dependent macropinocytosis; this uptake increases lysosomal activity and drives NLRP3 inflammasome assembly and IL-1β release, providing a direct pathophysiological bridge between mineral stress, the CaSR, and immune-mediated vascular injury (119, 131). Since CPP-triggered inflammation depends on CaSR signaling, positive allosteric modulation of the CaSR could in principle enhance CPP-induced NLRP3 activation in monocytes—an important consideration that parallels the CaSR–NLRP3 paradox discussed in Sections 3.3 and 6.1. Whether calcimimetics ultimately increase or decrease CPP-mediated inflammation *in vivo* is unresolved and clinically relevant. By lowering serum calcium, phosphate, PTH, and FGF23 (132), calcimimetics might possibly reduce the substrate available for CPP formation and improve calcification, plausibly diminishing CPP-driven monocyte activation in an indirect manner. This net effect, however, has not been tested directly and represents an untested but plausible hypothesis rather than an established action; the opposing possibility of enhanced CaSR-dependent CPP uptake cannot be excluded (133). Prospective studies measuring CPP load, T50, and monocyte IL-1β responses during calcimimetic therapy are needed to determine whether mineral-directed treatment translates into reduced CPP-associated inflammatory and vascular risk in CKD (134). In summary, the immune mechanisms discussed in this section—macrophage NLRP3 signaling and M1/M2 programming, Th17/Treg rebalancing, and CPP-driven inflammation—remain hypothesis-generating with respect to direct calcimimetic action in human CKD, as summarized in **Table 3**.

## 7 Vascular calcification: an osteoimmune and molecular perspective

### 7.1 Osteochondrogenic transdifferentiation of VSMCs: BMP-2–Runx2 and inflammatory pathways

Vascular calcification in CKD is an active, regulated, cell-mediated process in which vascular smooth muscle cells (VSMCs) lose contractile features and acquire osteochondrogenic phenotypes resembling bone-forming cells (135, 136). Hyperphosphatemia promotes VSMC calcification through sodium-dependent phosphate uptake, particularly via the type III phosphate transporter PiT-1, which induces osteogenic gene expression and mineral deposition in human vascular smooth muscle cells (52, 137). BMP-2 amplifies phosphate-induced VSMC calcification by increasing phosphate uptake, upregulating PiT-1, inducing Runx2, and suppressing smooth-muscle lineage markers such as SM22α (39). Runx2 is a key osteogenic transcription factor that drives VSMC phenotypic conversion and contributes to vascular calcification in experimental models (136, 138). Calcified VSMCs release mineralization-competent extracellular vesicles or matrix vesicles that can nucleate and propagate hydroxyapatite deposition, while tissue-nonspecific alkaline phosphatase promotes mineralization by degrading pyrophosphate, an endogenous inhibitor of vascular calcification (139, 140). Inflammatory macrophages, cytokines, oxidative stress, and macrophage- or VSMC-derived extracellular vesicles further amplify VSMC osteogenic conversion, creating a self-reinforcing inflammatory–calcific loop in CKD (141, 142).

### 7.2 CaSR signaling in VSMCs and calcimimetic anti-calcification mechanisms

Human vascular smooth muscle cells (VSMCs) express functional calcium-sensing receptor (CaSR), and reduced CaSR expression has been observed in calcified vascular tissue and in VSMCs undergoing mineralization (17). Experimental studies further show that calcimimetic exposure can increase CaSR expression, enhance CaSR trafficking to the plasma membrane, and markedly reduce calcium-induced mineral deposition in human VSMCs under pro-calcific conditions (84). These findings support a potential direct vasculoprotective effect of calcimimetics at the vascular wall, although the relative contribution of direct VSMC CaSR activation versus systemic changes in PTH, calcium, phosphate, and FGF23 remains incompletely defined (17, 84). Highly specific mechanisms, including CaSR-mediated suppression of BMP-2/Runx2 through Gαi/o–cAMP–CREB signaling, SP1-dependent upregulation of matrix Gla protein, or enhanced fetuin-A internalization through calreticulin-related pathways, should be considered mechanistic hypotheses rather than established calcimimetic actions (17, 84). Matrix Gla protein is a vitamin K–dependent inhibitor of vascular calcification; therefore, vitamin K status may influence vascular calcification risk and could modify the effectiveness of anti-calcification strategies in CKD (143–145). In the ADVANCE trial, cinacalcet plus low-dose vitamin D was associated with less progression of coronary artery calcium volume scores and some vascular/valvular calcification measures, but the primary Agatston coronary artery calcification endpoint did not reach conventional statistical significance; therefore, the vascular benefit should be interpreted cautiously (23, 24).

### 7.3 A unified bone–vascular–immune axis

CKD-MBD can be conceptualized as a self-reinforcing bone–vascular–immune disorder in which phosphate retention, secondary hyperparathyroidism, inflammation, abnormal bone remodeling, and vascular calcification interact through shared mediators, including phosphate, RANKL, Runx2, BMP-2, NF-κB, IL-1β, IL-6, and TNF-α (7, 146, 147). Within this model, calcimimetics primarily act by activating parathyroid CaSR, thereby suppressing PTH secretion and improving mineral-metabolic control (15, 19, 92). Experimental evidence also suggests that calcimimetics may have PTH-independent skeletal and vascular effects, because CaSR is expressed in bone cells and VSMCs, calcimimetic exposure can reduce VSMC mineral deposition, and calcimimetic treatment can maintain or improve bone parameters in PTH-clamped uremic models (18, 84, 95). In clinical studies, cinacalcet plus low-dose vitamin D attenuated progression of selected vascular and valvular calcification measures, although the primary coronary Agatston calcification endpoint in ADVANCE should be interpreted cautiously (23, 24). By contrast, proposed mechanisms such as direct FoxP3 stabilization, macrophage NLRP3 suppression, osteocyte Cx43-DAMP reduction, or FGF23 lowering through a defined osteocyte CaSR–ERK1/2–EGR1 pathway remain insufficiently validated and should be described as hypotheses rather than established human CKD mechanisms (24, 84, 95). Therefore, calcimimetics should be described as established PTH-lowering therapies with plausible PTH-independent skeletal and vascular actions, while their direct osteoimmune effects in human CKD-MBD remain investigational. The levels of evidence for the vascular mechanisms discussed in Section 7 are also summarized in **Table 3**.

## 8 Therapeutic implications and future directions

### 8.1 Clinical translation: established biochemical effects and potential osteoimmune mechanisms

To clarify the strength of the evidence discussed below, this section distinguishes the established biochemical effects of calcimimetics—suppression of PTH and improvement of calcium–phosphate control—from their investigational osteoimmune mechanisms. Reductions in FGF23, changes in bone–vascular biomarkers and proposed immunomodulatory actions such as NLRP3 inflammasome suppression and Th17/Treg rebalancing should be interpreted cautiously and regarded as hypothesis-generating pending further mechanistic and clinical validation. Calcimimetics have established clinical efficacy in dialysis-associated secondary hyperparathyroidism by suppressing PTH and improving calcium–phosphate biochemical control (87, 148). Clinical studies also show that cinacalcet and etelcalcetide can reduce circulating FGF23, although the mechanisms are likely multifactorial and may reflect changes in PTH, calcium, phosphate, vitamin D exposure, and bone turnover rather than a single direct osteocyte pathway (22, 28, 96, 148). Some small studies suggest that cinacalcet may influence selected bone–vascular biomarkers, including osteoprotegerin and fetuin-A, but these findings are exploratory and should not be interpreted as proven vascular-protective or osteoimmune mechanisms (149, 150). In the ADVANCE study, cinacalcet plus low-dose vitamin D was associated with attenuation of selected vascular and valvular calcification measures, although the primary coronary artery Agatston calcification endpoint did not reach conventional statistical significance (23, 24). Proposed osteoimmune mechanisms—including direct NLRP3 inflammasome suppression, Th17/Treg rebalancing, macrophage M1-to-M2 polarization, and restoration of B-lymphopoiesis—remain insufficiently validated as clinical effects of calcimimetic therapy in CKD; indeed, CaSR activation can promote NLRP3 activation in some macrophage models, and CKD-MBD–associated HSC niche defects were not restored by cinacalcet in one experimental study (79, 112, 116). Small clinical studies have examined inflammatory markers such as CRP and IL-6 during cinacalcet treatment, but the evidence is limited and inconsistent; therefore, calcimimetics should not currently be described as proven anti-inflammatory therapies (150–152). Future trials should incorporate integrated skeletal, vascular, immune, and clinical endpoints, including trabecular bone score, RANKL/OPG ratio, sclerostin, FGF23, IL-1β, IL-6, IL-18, Treg frequency, TRAP-5b, dp-ucMGP, vascular calcification progression, infection events, vaccine responsiveness, fracture incidence, and cardiovascular outcomes (**Table 4**).

### 8.2 Unresolved questions and controversies

A major unresolved question is the extent to which the skeletal and vascular effects of calcimimetics are mediated indirectly through PTH suppression versus directly through CaSR activation in osteoblasts, osteocytes, immune cells, or vascular smooth muscle cells. Experimental studies support direct skeletal effects of calcimimetic-mediated CaSR activation under PTH-clamped or uremic conditions, and *in vitro* data suggest that calcimimetics can increase CaSR expression and reduce mineral deposition in vascular smooth muscle cells (84, 95). Tissue-specific CaSR knockout models, including osteocyte-, macrophage-, and vascular smooth muscle cell–specific CaSR deletion in CKD-MBD models, would help determine whether these effects are cell-autonomous or secondary to systemic changes in PTH, calcium, phosphate, and FGF23 (153). Cinacalcet and etelcalcetide differ in chemical structure, route of administration, pharmacokinetics, and receptor-interaction mechanism, but no adequately powered CKD trial has prospectively compared their immune-cell, osteocyte, or vascular signaling profiles as primary mechanistic endpoints (88, 96). Since NLRP3 inflammasome signaling contributes to innate host defense and mTOR signaling regulates T-cell metabolism, any proposed long-term suppression of these pathways by calcimimetics should be evaluated carefully before immune-modulatory benefits are inferred clinically (116, 127). CaSR genetic polymorphisms may partly explain interindividual variability in cinacalcet response, and variants such as A986S and R990G/A990G should be incorporated into future pharmacogenomic analyses of calcimimetic efficacy and safety (154, 155).

### 8.3 Biased CaSR modulators and precision medicine

Biased CaSR modulation is an emerging pharmacological strategy in which ligands preferentially stabilize receptor conformations that favor selected G-protein- or β-arrestin-dependent signaling outputs, potentially allowing separation of desired PTH-lowering, skeletal, vascular, or anti-inflammatory effects from dose-limiting adverse events such as hypocalcemia or gastrointestinal intolerance (15, 61, 86). Recent structural and computational studies of CaSR, including cryo-EM analyses and large-library docking approaches, provide a framework for designing next-generation positive allosteric modulators with improved potency, receptor-state selectivity, and signaling bias (63, 156, 157). Combination therapy with calcimimetics and anti-sclerostin agents such as romosozumab is conceptually attractive for selected CKD-MBD phenotypes with high fracture risk and impaired bone formation, but this strategy remains unproven and requires careful evaluation of calcium–phosphate balance, bone turnover status, vascular calcification, and cardiovascular safety (158–161). To be clinically useful, biased CaSR modulation must specify the signaling profile that would separate parathyroid benefit from immune risk. In parathyroid chief cells, the therapeutic objective is efficient suppression of PTH, which is favored by G-protein–biased (Gαq/11 and Gαi/o) signaling (162). In monocytes and macrophages, however, these same Gαq/11- and Gαi/o-linked outputs promote CaSR-dependent NLRP3 inflammasome activation and IL-1β release (119). An ideal next-generation modulator would therefore exhibit G-protein–biased agonism in parathyroid tissue to lower PTH while avoiding Gq/Gi-driven inflammasome activation in myeloid cells—potentially by exploiting cell-specific receptor conformations, β-arrestin–biased signaling, or tissue-selective delivery—to prevent aggravation of macrophage-driven sterile inflammation and CPP-induced IL-1β release. Defining and validating such a bias profile is a prerequisite for any claim that a CaSR modulator can provide anti-inflammatory benefit without immune liability. On the other hand, SGLT2 inhibitors can attenuate NLRP3 inflammasome activity in cardiometabolic and kidney disease models, but synergy between SGLT2 inhibitors and calcimimetics for osteoimmune or CKD-MBD outcomes has not been clinically demonstrated and should be considered hypothetical (163–165). In future, using a precision-medicine approach, patients should stratify according to mineral, skeletal, vascular, inflammatory, and pharmacogenomic profiles—including PTH, calcium, phosphate, FGF23, bone turnover markers, RANKL/OPG ratio, sclerostin, dp-ucMGP, IL-6/IL-18, vascular calcification burden, and CASR genotype—and identify those most likely to benefit from specified calcimimetic regimens or future biased CaSR modulators (154, 155, 166, 167) (**Table 5**).

**Table 5:** Proposed molecular biomarker panel for osteoimmune phenotyping and therapeutic monitoring of calcimimetic therapy in CKD.

Category	Biomarker	Molecular pathway reflected	Clinical significance in CKD	Expected direction with calcimimetic therapy	References
Mineral metabolism					
Mineral metabolism	Serum PTH (intact)	Parathyroid CaSR–Gαi/o–↓cAMP–↓CREB–↓PTH gene transcription	Primary therapeutic target; correlates with high-turnover bone disease and RANKL upregulation	↓ (≥30–50% reduction expected; primary endpoint of all trials)	(21, 22, 148)
	Serum FGF23 (intact)	FGF23/FGFR-Klotho axis; reflects phosphate, vitamin D, PTH, and bone-turnover regulation.	High FGF23 is associated with cardiovascular and immune-relevant risk; mechanisms are multifactorial.	↓ validated; not a specific marker of direct osteocyte CaSR activation.	(57, 58, 96)
	Serum phosphate	Renal phosphate handling; VSMC Pit-1–ERK1/2 pro-calcific signaling; osteoblast apoptosis	Drives VSMC calcification; direct NLRP3 activation by phosphate should be interpreted cautiously and model-dependently.	↓ (mild; secondary to PTH suppression and dietary changes)	(41, 79, 118)
	1,25-Dihydroxyvitamin D (calcitriol)	CYP27B1 regulated by FGF23/Klotho; VDR–RXR → IL-10, ↓IL-12 (tolerogenic DCs); ↑intestinal Ca absorption	Low 1,25(OH)_2_D impairs VDR-mediated tolerogenic immune programming; deficiency common in CKD	Variable; may increase indirectly if FGF23 suppression improves CYP27B1 activity, but dialysis response is uncertain.	(5, 6)
Bone remodeling markers					
Bone remodeling	TRAP-5b (tartrate-resistant acid phosphatase 5b)	Osteoclast activity; reflects RANKL–RANK–TRAF6–NFATc1–cathepsin K pathway activation	High-turnover bone disease marker; predicts microarchitectural deterioration and fracture risk	↓ with reduced high-turnover activity; direct osteoclast apoptosis is not proven.	(11, 73)
	Bone-specific alkaline phosphatase (BSAP)	Osteoblast differentiation/matrix mineralization; reflects Wnt/β-catenin–Runx2–ALP pathway	Suppressed in adynamic bone disease; elevated in high-turnover disease; normalization indicates balanced remodeling	↓ (normalization of high-turnover; absolute anabolic effect on osteoblast function requires further study)	(95, 100)
	Serum sclerostin	Osteocyte SOST gene driven by MEF2C; Wnt/β-catenin inhibitor via LRP5/6 antagonism	Elevated in CKD; suppresses osteoblastogenesis and promotes adipogenesis; impairs fracture healing	Investigational; direct calcimimetic lowering of sclerostin via CaSR is not established.	(51, 76, 174)
	Serum DKK1	Osteocyte/stromal cell Wnt inhibitor; complements sclerostin in LRP5/6 antagonism	Amplifies Wnt/β-catenin suppression; correlates with vascular calcification; impairs bone marrow niche	Investigational; direct calcimimetic effect on DKK1 is not established.	(50, 51)
	RANKL/OPG ratio	Net osteoclastogenic drive; reflects balance between RANKL (osteoblast/T cell) and OPG decoy receptor	Elevated ratio predicts accelerated bone resorption, fracture risk, and vascular calcification in CKD	Possibly ↓ through PTH control and remodeling normalization; direct SP1-mediated mechanism unproven.	(11, 34, 70)
Inflammatory and immune biomarkers					
Innate immunity	IL-1β (serum/plasma)	NLRP3–ASC–caspase-1 cleavage product; NF-κB–driven pro-IL-1β precursor; IL-1R1–MyD88–IRAK1/4–NF-κB in target cells	Central driver of sterile inflammation; directly stimulates RANKL in osteoblasts (IL-1R1–NF-κB) and inhibits osteoblast function	Exploratory; direct calcimimetic-mediated NLRP3 suppression is not proven and may conflict with CaSR-NLRP3 data.	(116, 119, 127)
	IL-18 (serum/plasma)	Caspase-1 cleavage product from NLRP3 complex; amplifies IFN-γ from NK and T cells	Promotes Th1 over Treg; impairs adaptive immunity; correlates with CV mortality in HD patients	Exploratory; no established calcimimetic effect.	(116, 182)
	IL-6 (serum/plasma)	NF-κB–driven cytokine from macrophages/DCs; JAK1/2–STAT3 activator in T cells and osteoclasts	Drives Th17 polarization, osteoclastogenesis, FGF23 production in osteocytes, and acute-phase response	Inconsistent/exploratory; small studies only.	(34, 123, 177)
	TNF-α (serum/plasma)	NF-κB–mediated cytokine; TRAF2–NF-κB activation in osteoclast precursors; synergizes with RANKL	Amplifies RANKL-driven osteoclastogenesis; activates BMP-2 in VSMCs; drives M1 macrophage polarization	Exploratory; direct NF-kappaB/macrophage polarization mechanism unproven.	(115)
	hsCRP (high-sensitivity CRP)	Acute-phase reactant driven by IL-6–JAK–STAT3–hepatocyte response; surrogate of systemic NF-κB activation	Predicts CV events and mortality in CKD independently of traditional risk factors; reflects osteoimmune activation	Inconsistent/exploratory; not a validated anti-inflammatory endpoint.	(150, 152)
Adaptive immunity	Circulating Treg frequency (CD4^+^CD25^+^FoxP3^+^, % of CD4^+^)	FoxP3 stability (mTORC1–Ser418 phosphorylation); TGF-β–SMAD2/3; IL-2–STAT5 signaling	↓ Tregs associated with endothelial dysfunction, vascular inflammation, and impaired immune tolerance in CKD-HD	Exploratory; calcimimetic-mediated FoxP3/Treg restoration is unproven.	(122, 123, 177)
	Th17/Treg ratio (IL-17A^+^/FoxP3^+^ CD4^+^ T cells)	Balance governed by JAK-STAT3 (Th17) vs mTORC1–FoxP3/TGF-β–SMAD (Treg); metabolic state (glycolysis vs FAO)	Elevated Th17/Treg ratio predicts CVD, endothelial dysfunction, and poor vaccine responses in HD patients	Exploratory; proposed immune pharmacodynamic marker only.	(122, 123)
Vascular calcification markers					
Vascular calcification	Coronary artery calcification score (CACS) — CT Agatston	Cumulative VSMC transdifferentiation (BMP-2–Runx2) and macrophage-mediated mineralization; loss of CaSR–MGP inhibition	Strongest predictor of CV mortality in HD; tracks therapeutic impact on calcification biology	Attenuation of progression in selected measures; not reversal and not definitive outcome benefit.	(23, 146)
	Matrix Gla protein (uncarboxylated, dp-ucMGP)	Inactive MGP lacking vitamin K–dependent γ-carboxylation; reflects both MGP quantity and vitamin K status	↑dp-ucMGP predicts vascular calcification and CV mortality; low vitamin K diet common in CKD	Vitamin-K dependent; direct calcimimetic reduction of dp-ucMGP is unproven.	(167, 179, 184)
	Fetuin-A (α2-Heremans–Schmid glycoprotein)	Circulating calcification inhibitor forming calciprotein particles (CPPs); CaSR–ERK1/2 promotes VSMC internalization	↓ Fetuin-A in CKD predicts CV calcification risk; calciprotein particles trigger TLR4/NF-κB on macrophages	Uncertain; direct CaSR-mediated fetuin-A internalization is not established clinically.	(84, 185)
Hematopoietic niche	Lymphocyte-to-monocyte ratio (LMR)	General immune/inflammatory status; CKD-MBD niche disruption; not validated as CaSR-dependent CXCL12/SCF restoration.	Low LMR reflects myeloid skewing; associated with infection risk, immune senescence, and inflammation in dialysis	Exploratory; cinacalcet did not restore CKD-MBD HSC defects in one experimental model.	(36, 60, 114)

PTH, calcium, phosphate, FGF23, bone-turnover markers, and vascular calcification are the strongest endpoints; immune biomarkers should currently be treated as exploratory research endpoints.

## 9 Conclusion

The convergence of osteoimmunology and CKD-MBD has highlighted the calcium-sensing receptor (CaSR) as a biologically important mineral-sensing receptor that may connect parathyroid function, skeletal remodeling, vascular calcification, and selected immune-cell responses. Calcimimetics were developed primarily to treat secondary hyperparathyroidism by activating parathyroid CaSR, thereby lowering the calcium set-point for PTH secretion and improving calcium–phosphate biochemical control in dialysis patients. Beyond their established endocrine actions, experimental studies suggest that calcimimetics may exert PTH-independent skeletal effects, including maintenance of bone turnover and stimulation of osteoblast-related bone formation under uremic or PTH-clamped conditions (95). Additional experimental and clinical data indicate that calcimimetics may influence vascular calcification through improved mineral metabolism and possible direct vascular CaSR-dependent mechanisms in VSMCs, although the relative contributions of systemic versus direct vascular effects remain incompletely defined (17, 24, 85). Calcimimetics also reduce circulating FGF23 in dialysis patients, but this effect is likely multifactorial and should not yet be attributed solely to a direct osteocyte CaSR–ERK1/2–EGR1 pathway (27, 28, 96). In contrast, proposed immune mechanisms—including direct NLRP3 inflammasome suppression, macrophage M1-to-M2 polarization, Treg/Th17 restoration, FoxP3 stabilization, and recovery of CXCL12/SCF-dependent lymphopoiesis—remain insufficiently validated as clinical effects of calcimimetic therapy in CKD (79, 112, 116). Therefore, calcimimetics should currently be viewed as established PTH-lowering therapies with plausible PTH-independent skeletal and vascular actions, while their direct osteoimmune effects remain an important but still investigational area. Future research should prioritize tissue-specific CaSR models, head-to-head mechanistic comparisons of cinacalcet, etelcalcetide, and evocalcet, and biomarker-guided clinical trials integrating PTH, calcium, phosphate, FGF23, bone turnover markers, vascular calcification burden, inflammatory biomarkers, and CASR genotype. Ultimately, a cautious CaSR-centered osteoimmune framework may help guide the next generation of calcimimetic research, including biased CaSR modulators, rational combination strategies, and precision-medicine approaches designed to improve skeletal integrity and cardiovascular resilience in CKD-MBD without overextending current evidence.
